# The influence of stakeholder interests on safety outcome reporting in psychedelic research and implications for science communication

**DOI:** 10.47626/2237-6089-2024-0866

**Published:** 2025-04-07

**Authors:** Elena Koning, Marco Solmi, Elisa Brietzke

**Affiliations:** 1 Centre for Neuroscience Studies Kingston ON Canada Centre for Neuroscience Studies (CNS), Kingston, ON, Canada.; 2 Queen's University School of Medicine Department of Psychiatry Kingston ON Canada Department of Psychiatry, Queen's University School of Medicine, Kingston, ON, Canada.; 3 University of Ottawa Department of Psychiatry SCIENCES Lab Ottawa ON Canada SCIENCES Lab, Department of Psychiatry, University of Ottawa, Ottawa, ON, Canada.; 4 The Ottawa Hospital Department of Mental Health Regional Centre for the Treatment of Eating Disorders and On Track Ottawa ON Canada Regional Centre for the Treatment of Eating Disorders and On Track: The Champlain First Episode Psychosis Program, Department of Mental Health, The Ottawa Hospital, Ottawa, ON, Canada.; 5 Clinical Epidemiology Program University of Ottawa Ottawa Hospital Research Institute Ottawa ON Canada Ottawa Hospital Research Institute (OHRI) Clinical Epidemiology Program University of Ottawa, Ottawa, ON, Canada.; 6 Charité Universitätsmedizin Department of Child and Adolescent Psychiatry Berlin Germany Department of Child and Adolescent Psychiatry, Charité Universitätsmedizin, Berlin, Germany.

**Keywords:** Psychedelics, conflicts of interest, safety, clinical trials, science communication

## Abstract

Psychedelics are a group of psychoactive substances that produce complex and subjective changes to consciousness and carry unique safety considerations. There is a growing body of work investigating the use of psychedelics in mental health treatment alongside increasing socio-cultural and political acceptance. This rapid evolution has prompted corporations to fund psychedelic clinical trials, leading to a potential rise in conflicts of interest in relevant studies and publications. However, the body of evidence for the safety and efficacy of psychedelic-assisted psychotherapy is early. There is concern regarding the introduction of bias in psychedelic clinical trials and the selective reporting of results amidst and beyond corporate involvement. At a crucial time in psychedelic drug reform, this paper explores the safety concerns associated with psychedelics, the potential influences of financial stakeholders on safety outcome reporting and the importance of balanced science communication in maintaining public health and safety.

## Introduction

Psychedelics are a group of naturally occurring and synthetic psychoactive substances that produce complex and subjective changes to consciousness. The classical psychedelics include lysergic acid diethylamide (LSD), dimethyltryptamine (DMT) and psilocybin which act primarily as an agonist at 5-HT_2A_ receptors. Conversely, 3,4-methylenedioxymethamphetamine (MDMA) is a non-classic psychedelic and non-selectively promotes monoamine receptor activation. Ketamine is a dissociative anesthetic and acts as an antagonist of the N-methyl-D-aspartate (NMDA) receptor. Although chemically distinct, the perceived effects of psychedelics are relatively similar, including marked changes in perceptual, cognitive and affective domains. For example, the stimulation of 5-HT_2A_ cortical layer V pyramidal neurons by classic psychedelics triggers disruptive changes to cortical connectivity, leading to a temporary state of reduced cognitive rigidity, increased interoception and improved mood.^1^ These changes provide an opportunity to overcome maladaptive thinking patterns when integrated into a therapeutic framework, becoming an emerging tool of interest in psychiatry and psychotherapy.

Mental health issues impact a significant and increasing proportion of the global population and pose a substantial burden at both individual and societal levels.^2^ Conventional treatment approaches are not effective in all cases, while psychedelic-assisted therapy has demonstrated promising preliminary results. For example, studies of the classic psychedelics have demonstrated evidence of safety and therapeutic efficacy for symptoms of depression and anxiety.^3-6^ Similarly, ketamine has been found to induce rapid and long-lasting antidepressant effects.^7-10^ MDMA-assisted psychotherapy has demonstrated high rates of tolerability, clinical response and remission in individuals with PTSD symptoms.^11^

Alongside promising research outcomes, there is an increasing socio-cultural acceptance, including a growing presence of psychedelics in the media. For example, popular Netflix documentaries such as "How to Change Your Mind" portray ineffable and ‘life-changing’ effects after a single dose. Although public narratives are commonly sensationalized, these shifting public opinions have counteracted the stigmatization that occurred in the mid-twentieth century when psychedelics were classified as having no or very little medical purpose, a high potential for abuse and a lack of accepted safety.^12^ Today, the decriminalization of psychedelics may occur across North America. For example, 25 U.S. states have considered 75 psychedelic reform bills with 10 enacted and 32 still active.^13^

In concert with increasing public and political acceptance of psychedelics, there has been substantial economic growth of the psychedelic research and development sector over recent decades.^14-16^ The psychedelic drugs market is projected to be worth nearly $12 billion USD by 2029, outpacing the cannabis market.^17,18^ While early research on psychedelics was supported through academic or philanthropic means, the growing financial interest in psychedelics has prompted pharmaceutical companies to fund clinical trials. For example, in 2020 and 2021 alone, venture capital investments totaled $31.2 million, far greater than contributions from non-profit or public funding agencies.^19-21^ Further, some companies are seeking exclusive rights to psychedelic access in medical contexts and there is a rapid increase in the number of patents. For example, synthetic proprietary formulations of psilocybin are emerging, such as COMP360 owned by Compass Pathways.^22^ Combination drugs and even the context in which they are being administered have been patented.^23^

Despite the financial excitement, evidence for the safety and therapeutic efficacy of psychedelic substances in humans has not been fully explored, with the majority of research being conducted with methodological challenges that threaten validity.^24^ Corporate involvement has catalyzed the development of psychedelic therapies and has contributed to further understanding. However, it had also led to a rise in the conflicts of interest (COIs) associated with relevant studies and publications. COIs spark concern over the introduction of bias from the earliest stages of study design to the reporting of results. For example, studies reporting COIs are five times more likely to report positive results than studies not reporting COIs.^25^ In particular, there is concern that the financial exuberances will contribute to over-promising the benefits of psychedelic medicines while undermining the potential safety risks.

Corporate interests, as well as the pressure to publish positive results in academia, do not only influence how psychedelic research is studied and implemented but may also how the results of clinical trials are communicated to the public. Scientific findings are often sensationalized in the media, including selective reporting of positive findings, undermining safety concerns and sharing enthusiastic stories of transformation. These over-exuberant and biased narratives may pose a risk to the public considering the unique safety concerns associated with psychedelics and lack of evidence for safety and efficacy outside of controlled, medical contexts. Despite these risks, the influence of COIs in psychedelic safety outcome reporting remains relatively unexplored. Corporate involvement in psychedelic therapy is inevitable and it is important to consider the appropriate measures to limit the negative effects while maintaining the benefits. The objective of this paper is to contribute to the discussion, including an overview of the safety of psychedelics, how stakeholder interests can influence safety outcome reporting and the importance of science communication in promoting awareness and safe use.

## Safety concerns associated with psychedelics

Psychedelic-assisted psychotherapy is the primary framework in which psychedelic research is conducted today, involving a high-dose administered as an adjunct to psychological support or psychotherapy, with preparation and integration sessions occurring before and after dosing, respectively. The environment in which the experience occurs is an important consideration and psychedelic therapy is often carried out in a comfortable living room-like setting with calming music and an aesthetically pleasing ambience. Low-dose psychedelic interventions are also being investigated (i.e., ‘microdosing’) and have the potential to be integrated into the standard model of pharmacotherapy for psychiatric illnesses as it does not typically require the intense supervision and psychological support associated with high dose sessions. However, the safety and therapeutic evidence for microdosing is primarily limited to preclinical data as most clinical trials use high doses.^26-30^ The current literature generally considers psychedelics safe to consume in the appropriate population and under controlled, medically supervised environments.^12,31-35^ Nonetheless, psychedelics are associated with a range of safety concerns, many of which are not typically associated with other pharmaceuticals.

The specific safety profile and adverse effects of psychedelics vary depending on the substance and dosage. Psychedelics are considered relatively physiologically safe when administered at standard doses; typically up to 10-25 milligrams of psilocybin, 80-120 milligrams of MDMA, 50-90 milligrams of ketamine and 50-200 micrograms of LSD.^36-38^ Administration is typically oral for classical psychedelics and MDMA, while intranasally or intravenously for ketamine. The duration of effects of psychedelics is dose-dependent, but usually lasts about 1 hour for ketamine, 4-6 hours for psilocybin and MDMA, while LSD may last up to 12 hours.^39^ The effects of psychedelics were not associated with sex or body weight in a pooled sample, although genetic polymorphisms of the CYP2D6 enzyme have been shown to significantly influence pharmacokinetics and the subjective effects of LSD.^40^ Low-dose studies are typically conducted with psilocybin and involve 0.5-3 micrograms per kilogram which does not typically induce perceptual changes.^26^

Overdose with psychedelics is rare and is typically reported when mixed with other substances such as alcohol.^41,42^ For example, eight individuals who took 1000-7000 ug/100 ml of LSD experienced comatose, hyperthermia, vomiting, light gastric bleeding and respiratory issues, but made a full recovery without residual effects.^43^ The median lethal dose of psilocybin was determined to be 280 mg/kg in rats.^44^ Although deaths due to psychedelic overdose are not well-documented in humans, the effects of psychedelics have contributed to injury and death in unsupervised settings.^45-47^ These cases highlight the intense perceptual and cognitive changes that occur under the influence of psychedelics and the importance of professional supervision during these experiences. Another concern is the potential for interaction between psychedelic substances and other medications. For example, the co-administration of psychedelics, especially MDMA, with serotonin reuptake inhibitors has been associated with the development of serotonin toxicity, although more work is needed to understand this phenomenon.^48^ Conversely, serotonergic psychedelics may not achieve the desired effects in individuals taking antipsychotics such as quetiapine or olanzapine which block the 5-HT_2A_ receptor, risking symptom worsening or reactions if participants discontinue medication.^49^

Regarding abuse, there is limited risk of addiction or dependence shown in humans and animals as the mechanistic actions of serotonergic psychedelics do not directly act on the mesolimbic dopaminergic system.^31^ Hallucinogen use disorders refer to the dependence and abuse of hallucinogenic substances leading to clinically significant impairment or distress. The majority of individuals do not exhibit hallucinogen use disorders after psychedelics use, suggesting a low risk for abuse.^50,51^ Psychedelics typically demonstrate the lowest rate of abuse when compared to other drugs and are repeatedly shown to not cause withdrawal symptoms, dependence of compulsive use.^12,31,52,53^ Tolerance, including cross-tolerance, has been documented in relation to the euphoric and perceptual effects of psychedelics, but not the somatic effects.^34,54,55^

Most of the acute side effects reported during high-dose experiences with psychedelics include transient, delayed headache of dose-dependent severity, nausea, vomiting and cardiovascular changes.^31,36^ For example, psychedelics may induce sympathomimetic effects, including vascular smooth muscle contraction, increased heart rate, blood pressure, pupil dilation and increased body temperature, changes which normalize within 24 hours of administration.^40,56-60^ Delayed headache may begin 7 hours following drug administration, but is typically not severe or disabling.^61^

Psychedelics are associated with a range of psychological risks, the most common of which are challenging experiences involving anxiety, paranoia and/or confusion.^62^ Challenging experiences are typically transient, improved with psychological support and have been shown to correlate with improved therapeutic outcomes after the psychedelic session.^40,56,63-66^ For example, although 39% of respondents rated challenging experiences under psychedelics to be in the top five most challenging experiences of their life, the degree of difficulty was associated with the degree of increased well-being after the experience.^64^ Interestingly, Aday et al. found an association between personality traits of absorption, openness and acceptance with positive psychedelic experiences while individuals who were preoccupied or apprehensive were more likely to experience acute adverse effects.^67^ Rescue from experiences that are too distressing can be accomplished with benzodiazepine or a 5-HT_2A_ receptor blocker such asolanzapine.^49,61,68^ Symptoms of transient anxiety typically dissipate upon interpersonal support and no residual effects were found in eight double-blind placebo-controlled studies.^69,70^

More severe psychological effects of psychedelics are far less common, especially in non-clinical populations.^5,12,71^ Hallucinogen persisting perception disorder (HPPD) is defined as a long-lasting condition characterized by spontaneous recurrence of visual disturbances reminiscent of acute hallucinogen intoxication. HPPD may include perceptual movement and geometric shapes, blurring of patterns, halo effects, after images and macro- and micropsia, causing significant impairment. Although the incidence of HPPD is not known, few cases of HPPD have been documented following psychedelic use, particularly LSD.^31,72^ The American Psychiatric Association reported a prevalence of HPPD in 4.2% of hallucinogen users, although this rate is expected to be much lower in clinical contexts with appropriate screening, preparation and dosing procedures.^61,73^ A recent systematic review of 16 clinical trials evaluated the safety and efficacy of psilocybin and found zero cases of psilocybin-induced psychosis or HPPD.^62^

Psychological vulnerability is another safety concern as psychedelics profoundly alter the way individuals think and perceive their environment. For example, there is evidence suggesting that participants under the influence of psychedelics tend to be more agreeable and open.^74-76^ In this way, participants may be more suggestible during psychedelic experiences and therapist talk engagement should occur only when initiated by the participant. There is also a risk for therapist abuse and sexual misconduct, especially with MDMA which is known to impact desire for emotional and social intimacy.^77,78^ There remains limited oversight or specific guidelines on the therapist-patient boundary in psychedelic-assisted therapy, however, it now typically occurs in the presence of a pair of therapists, video recording of the sessions, and therapists training.^12,79^

The safety concerns and risks associated with psychedelics have largely been incorporated into the modern clinical trials and practice today with recommendations provided in numerous published guidelines.^61^ For example, appropriate screening, preparation, dosing and psychological support are deemed critical. Pregnant or breast-feeding women and individuals with uncontrolled hypertension are typically excluded due to the cardiovascular effects of psychedelics.^61^ Despite these measures, there is a lack of proper dose response understanding for psychedelics which may mitigate safety risks further.^80^ There is also the problem of informed consent in which the ineffable nature of the psychedelic experience makes it difficult to fully inform participants of the risks. Informed consent in psychedelic studies should include what all procedures will entail, benefits and risks, the long-lasting impacts and any alternative options.

While generally considered safe according to the existing literature, the mechanistic underpinnings and long-term effects of psychedelic substance use remain to be fully delineated. Few studies have evaluated the risks associated with the use of psychedelics in the long-term as follow ups are typically conducted at 6-12 months.^81^ Cross-sectional studies have indicated abnormalities in regular psychedelic users. For example, regular ayahuasca users scored significantly higher on neuropsychological tests when compared to matched controls, although cortical thinning was found in the middle frontal gyrus, inferior frontal gyrus, precuneus, superior frontal gyrus and posterior cingulate cortex. Interestingly, cortical thickening was found in the precentral gyrus and anterior cingulate cortex in ayahuasca users.^82^ Arguably the largest safety concern associated with psychedelics is their recreational use which can occur in the absence of proper screening, preparation and integration sessions, and in the absence of trained medical and psychological support. For example, the expectations of the participants (i.e., set) and the physical and social environment in which the experience takes place (i.e., setting) likely contribute to adverse events during a psychedelic experience and are not regulated outside of clinical settings. Together, the aforementioned safety concerns highlight the importance of thoughtfully designed psychedelic clinical trials, transparent reporting and proper communication of risks and safety data to the public.

## Financial conflicts of interest in safety outcome reporting

As data emerge for the safety and efficacy of psychedelics in psychiatry, it is important to scrutinize the landscape in which the research is conducted, including the influence of COIs. Psychedelic clinical trials are increasingly funded by large corporations, increasing concerns over the potential impact of stakeholder interests. There are numerous ways in which COIs may influence safety outcome reporting, as summarized in Table 1.

**Table 1:** The main sources of financial conflicts of interest in psychedelic research that impact safety outcome reporting

Area of research	Conflict of interest
Research topic selection	Corporations may prioritize research fields related to products, processes or activities that can be best commercialized
Investigator psychology	Researchers may experience social and professional pressure to conform with corporate expectations
Substance selection	Corporate interests may favor substances with lower risks of adverse outcomes
Population selection	Corporate interests may favor study populations with a lower risk of adverse outcomes
Study design	Corporate interests may favor study designs that do not identify adverse events and/or do not have appropriate follow-up
Cost minimization	Corporate interests may favor reduced monitoring, sessions, and less frequent follow-ups or open interviews with participants to reduce costs
Reporting	Selective reporting and publication bias may affect industry and non-industry funded reports
Media engagement	Media reports are frequently sensationalistic and propose narratives that are not balanced
Participant psychology	Selective reporting in the media may contribute to excessive enthusiasm and expectancy bias in participants

As any for profit entity, corporations are known to prioritize research fields related to products, processes or activities that can be commercialized, which can lead to the redirection of clinical research fields away from public health interests and towards financial interests. In the field of psychedelic medicines, this may involve certain indications and products being researched due to their marketability instead of therapeutic efficacy.^83^ For example, many clinical trials are evaluating the safety and efficacy of psilocybin-related synthetic analogs because unprocessed psilocybin mushrooms are naturally-occurring substances and, therefore, cannot be patented. The company Mydecine Innovations Group developed a proprietary compound MYCO-005, which mimics the active ingredient in psilocybin, but with a more rapid onset and shorter duration of action.^84^ In this way, corporations may steer the conduct of psychedelic trials towards profit-driven research topics, potentially away from topics with the most therapeutic potential. However, this is not always the case as corporate funding has allowed for highly impactful therapeutic options to be developed and accelerated into the public domain. The same may be true for the interests of non-profit organizations.^85^ Regarding safety outcomes, corporations may be more likely to fund clinical indications with a lower risk for adverse outcomes, not necessarily those that provide the widest public health benefit.

To understand how stakeholders may influence safety outcome reporting in psychedelic studies, it is important to consider the relationship between corporate funders and research teams. Motivational biases on the part of corporations may influence decisions made by scientists in the conduct of research, through social and institutional pressures. Stakeholders may take part in providing ‘gifts’ to build a relationship with scientists, such as the invitation to talk at a sponsored event, providing meals and covering costs of travel or accommodation.^86^ Further, corporations are not the only source of bias influencing investigator psychology. Scientific journals promote a concept colloquially referred to as ‘publish or perish’ by favoring positive over negative results in the publication process. In this way, researchers may experience a pressure to conform with corporate expectations, produce positive results, and confirm pre-existing beliefs, whether conscious or not. Therefore, even if decisions are being made by scientists, they are not separate from the influence of financial COIs and publication bias.

Corporate interests may also influence the methodological design of psychedelic clinical trials, similarly, favoring designs that lead to favorable safety outcomes. This may include the implementation of strict selection criteria that excludes individuals at a high risk of experiencing adverse events. A lack of reasonable screening may reduce the generalizability of the results, as individuals in the target population are unnecessarily excluded. In this way, participants of small psychedelic studies may be easier to treat.^24^ Further, psychedelic studies mainly include Caucasian individuals of socioeconomic stability which is also detrimental to generalizability.^87-89^ Design decisions may also include those which favor statistical validity. For example, a pharmaceutical company-funded trial of psilocybin for treatment-resistant depression registered the trial period of "up to 12 weeks," allowing for alterations to the statistical significance to be made post-trial.^90^

Corporations have the priority to maximize returns on investment and, as a result, favor the minimization of costs associated with psychedelic products and the clinical trials surrounding them. Industry involvement in trial design may contribute to the minimization of safety procedures as a result of the motivation to cut costs. Preparation, dosing and integration sessions are lengthy in nature and require the presence of paid healthcare professionals. Therefore, corporate interests may favor reduced resources for monitoring and oversight during clinical trials, including a smaller sample size and less frequent assessments or follow-ups.^71^ In this way, microdosing is an appealing avenue for further investigation, despite limited evidence for therapeutic efficacy.^91^ A lack of adequate follow-up assessment may contribute to an inaccurate representation of the safety profile associated with an intervention, as adverse events occurring after the study are not captured or reported. It is known that specific questioning of study participants leads to the reporting of more adverse effects when compared to more general questioning. In this way, streamlined study designs may not prioritize open interviews and structured rating procedures, leading to adverse events going unreported.^92^ While corporations may favor study designs that minimize costs and limit the identification of adverse events, this may also be an expectation of the investigators due to publication bias and limited funding availability.

Motivational biases arising from corporate involvement in psychedelic research may also impact the accurate reporting of safety outcomes through the promotion of selective reporting. For example, a psychedelic-assisted psychotherapy trial found increased suicidal ideation and self-injury in experimental groups (10 and 25 mg), while the control group (1 mg) did not. The authors minimized these adverse results in a media report indicating that they were "probably random events unrelated to the dose of psilocybin."^90,93^ In another trial, 4 of 15 participants that took ayahuasca had to be hospitalized, while authors wrote that the study found "safety and therapeutic value in psychedelics."^94^ Publication bias and selectively reporting of positive results contributes to the inflation of effect sizes upon meta-analysis and underestimates safety risks.^24^

Stakeholders may also influence clinical trial reporting through the media, both directly and indirectly. Incomplete reporting of safety and efficacy data and an emphasis on positive results may skew the public perception of psychedelic medicines. This is exacerbated even more by sensationalized Netflix documentaries, psychedelic tourism and related influencers on social media. While corporations favor positive narratives of psychedelics, this is also a bias held by news and media platforms which profit from sensationalist narratives. In clinical research, this may contribute to expectancy bias, a phenomenon in which preconceived beliefs held by stakeholders, researchers and participants influence study results.^95^

The term ‘excessive enthusiasm’ has been used to describe the highly optimistic opinion of the therapeutic potential of psychedelics and may be detrimental to the accurate reporting of safety outcomes. For example, participants who hold positive views associated with psychedelics or who have previous experiences with these substances may be more likely to participate in psychedelic clinical studies, to identify the effects of psychedelics (i.e., ‘break the blind’) and expect positive outcomes. Conversely, individuals who have had negative experiences with psychedelics may be less likely to participate, creating a self-selecting study population that reduces the likelihood of adverse events.^58,67^ It is also possible that participants do not want to report poor outcomes to avoid impacting access to individuals that are reporting positive results from psychedelic therapy.^24^ ‘Excessive enthusiasm’ also extends to researchers, in which those with positive beliefs about psychedelics may be more likely to study them, expect and emphasize positive outcomes and be less likely to identify negative outcomes, including adverse events.

Together, there are numerous threats to the validity of psychedelic studies as related to stakeholder involvement, including in the design, conduct and reporting of clinical trials. At worst, the influence of COIs may add to the confluence of factors leading to premature legislative changes. For example, there is a documented underreporting of adverse events related to esketamine in individuals with depression.^96^ A 2021 meta-analysis concluded that there is insufficient safety data for ketamine, despite FDA approval of nasal esketamine for treatment-resistant depression in 2018. This is not the first time a commercial industry has contributed to a "regulatory vacuum" and public health risks.^86^ For example, it has been estimated that hundreds of thousands of deaths could have been prevented had stricter regulations on the commerce of tobacco products been enacted when proposed by scientists in the 1960s.^97^ More recently, the relationships garnered between prescribers and the opioid industry has posed a significant harm to the public, especially marginalized groups.^98^ While the psychedelic industry is unique from those of tobacco and opioids, caution is still warranted regarding legislative changes that may facilitate recreational use and abuse.

Promoting alternative forms of research funding and restricting corporate control over psychedelic research may help avoid over commercialization of these substances. Goldberg et al.^86^ argued that disclosure of COIs is not enough to mitigate the associated health risks of corporate involvement, but rather the elimination of relationships between commercial industry and scientists (i.e., ‘sequestration’) is the optimal solution. Disclosure does not interfere with the motivational bias and relationships built between industry and scientists and there is no evidence to support its efficacy in promoting partiality.^86,99-101^ Disclosure may even intensify the negative consequences of COIs, fueling distrust between scientists and the public in some cases. Even at the level of evaluation of COIs, individuals with a personal interest in the area of conflicted research provided more lenient evaluations of researcher COIs in a 2018 analysis.^102^ While sequestration may eliminate the impact of COIs, this may not be the ideal solution. Financial interests accelerate the development of therapeutic options, and it is arguable whether the benefits of corporate involvement outweigh the risks.

## Promoting balanced science communication

As corporate involvement in psychedelic research will likely persist, it is important to consider approaches that counteract the biases associated with COIs and promote a balanced public perception of the field. Historically, opinions about psychedelics have been skewed by media reports, including selective reporting and excessive fearmongering. For example, there was a disproportionate media coverage of individuals jumping from buildings or committing suicide under the influence of psychedelics. Paradoxically, these outcomes more often occur in individuals under the influence of alcohol.^12^ More recently, the opposite may be true, in which the therapeutic effects of psychedelics are more likely to be discussed in the media in a sensationalized manner when compared to the safety concerns. For example, misleading yet recurring themes in the media include the ‘loosening’ of restrictions on psychedelic access, the ability of psychedelics to ‘cure’ mental health issues, the efficacy of ‘micro-dosing’ and the ability of psychedelics to replace traditional therapeutic models.^80^

The degree of media attention that psychedelics have garnered is unusual for substances at this level of research and development. This spotlight can be attributed to, not only the cultural and political significance of psychedelics in the mid-twentieth century, but also their association with ceremonial and therapeutic use by indigenous communities for centuries. Further, their status as ‘natural’ or ‘plant-derived’ products, particularly psilocybin, could lead to the commercialization of psychedelics as wellness products opposed to a medical tool for specific indications.^80^ This may lead individuals to believe that psychedelics are safe, a position that is only amplified in social media communities, despite lack of scientific support.^80^ Yaden et al. described the "The Gartner Hype Cycle" in which novel advancements trigger substantial attention, leading to inflated expectations followed by a steep decline when expectations are not met. In this way, public narratives may shift from overly positive to overly negative.^103^ Scientists have a responsibility to dispute unsupported claims to reduce the harms associated with such a polarizing social pattern.

Balanced science communication is essential to foster a more critical and informed interpretation of the safety of psychedelics and can be accomplished through several avenues in both public and scientific communities. Media outlets should present information responsibly, including a balanced discussion of potential risks and limitations, opposed to sensationalizing positive results. Media guidelines and regulatory oversight could be developed and incorporated for reporting sensitive topics such as psychedelic-assisted psychotherapy. The promotion of media literacy is another important avenue. Conducting media literacy initiatives to educate the public about biases in science reporting equips individuals with the skills to critically evaluate media coverage, discern levels of evidence, sources and recognize the implication of COIs.^104^ For example, a meta-analysis of 51 media literacy interventions found positive effects on outcomes of media knowledge, criticism, perceived realism, influence, behavioral beliefs, attitudes, self-efficacy and behavior.^105^ Improved media literacy will empower the general public with a more nuanced and evidence-based approach to media consumption.

Researchers hold responsibility in the accurate communication of findings to media outlets, although public knowledge translation is not typically part of standard graduate training. Programs have been created to improve the public speaking, science journalism and teach capabilities of graduate students and should be more widely employed.^106-108^ In the scientific publication process, while Goldberg et al.^86^ described the inadequacy of disclosing COIs, transparency in this way contributes to the awareness of potential biases associated with the research. One step further, researchers could pre-register study protocols and outcomes before the acquisition of funding and initiation of research, including the transparent disclosure of exclusion criteria, recruitment procedures and follow-ups. This may help overcome selective reporting of positive outcomes and reduce the influence of hindsight biases. Journals should prioritize systematic over narrative reviews to avoid tones of ‘excessive enthusiasm,’ including a summary of safety outcomes as a requirement of systematic reviews and meta-analyses on the topic.

It is important to promote diversity in research teams and participants, including those with and without previous psychedelic experiences. This will help minimize biases and promote generalizability among results, contributing to more valid reporting of safety outcomes. Further, researchers should include data on safety and adverse events as a primary or secondary outcome of psychedelic studies, not in the supplementary materials section, which occurs in a substantial number of reports.^24^ In addition, reviewers and journals should be more stringent in the reporting of safety outcomes, including a comparison of clinical trial results with published protocols. There is currently no definition of an adverse event in psychedelic research or standardized measurements.^24^ While there are the Consolidated Standards of Reporting Trials, these are not always enacted in clinical research. It may be helpful to include independent arbiters to determine whether adverse events are related to the treatment, as suggested by van Elk et al.^24^ This will play a critical role in evaluating the safety of interventions as well as the ethical and methodological rigor of trials to minimize the influence of stakeholders and promote balanced communication of safety outcomes.

## Conclusion

Psychedelics are powerful substances with the potential to induce both harm and good at individual and societal levels. There is a need for increased awareness regarding the impact of financial and stakeholder interests on safety outcome reporting associated with psychedelics. Increased transparency, communication and a consideration for the influence of stakeholders may stimulate more public funding in the field and reduce COIs in psychedelic research and reporting. Conversely, corporate involvement has and continues to catalyze advancements. In the meantime, ethical science communication and public education will help combat misinformation and contribute to the safe use of psychedelics amongst the public. Ultimately, while market-driven solutions have contributed to the development of life-changing therapies, more awareness is needed concerning how the psychedelic industry may threaten research integrity and pose public safety risks in the pursuit of profit.
